# SMARCB1/INI1 germline mutations contribute to 10% of sporadic schwannomatosis

**DOI:** 10.1186/1471-2377-11-9

**Published:** 2011-01-24

**Authors:** Guillaume Rousseau, Tetsuro Noguchi, Violaine Bourdon, Hagay Sobol, Sylviane Olschwang

**Affiliations:** 1 Neuropediatrics Department, Children Hospital, Montreal, Canada; 2 Laboratory of Molecular Oncogenetics, Institut Paoli-Calmettes; Marseille, France; 3 Université La Méditerranée; Marseille, France; 4 Centre de Recherche en Cancérologie de Marseille, Institut Paoli-Calmettes, 232 boulevard Sainte-Marguerite, 13009 Marseille, France

## Abstract

**Background:**

Schwannomatosis is a disease characterized by multiple non-vestibular schwannomas. Although biallelic *NF2 *mutations are found in schwannomas, no germ line event is detected in schwannomatosis patients. In contrast, germline mutations of the *SMARCB1 *(*INI1*) tumor suppressor gene were described in familial and sporadic schwannomatosis patients.

**Methods:**

To delineate the *SMARCB1 *gene contribution, the nine coding exons were sequenced in a series of 56 patients affected with a variable number of non-vestibular schwannomas.

**Results:**

Nine variants scattered along the sequence of *SMARCB1 *were identified. Five of them were classified as deleterious. All five patients carrying a *SMARCB1 *mutation had more multiple schwannomas, corresponding to 10.2% of patients with schwannomatosis. They were also diagnosed before 35 years of age.

**Conclusions:**

These results suggest that patients with schwannomas have a significant probability of carrying a *SMARCB1 *mutation. Combined with data available from other studies, they confirm the clinical indications for genetic screening of the *SMARCB1 *gene.

## Background

Neurofibromatoses (NF) are an heterogeneous group of genetic disorders predisposing to various tumors of the nervous system, divided into two well recognized distinct clinical entities, NF1 and NF2. Patients with multiple non-vestibular schwannomas have been assembled into a particular category within neurofibromatoses called schwannomatosis [1]. The *SMARCB1 *gene has been found to harbor germline alterations in both familial and sporadic schwannomatosis patients [2,3], with a greater number of spinal schwannomas in familial cases and the presence of meningiomas [4] although this latter point remains debated [5]. To improve the clinical indications for *SMARCB1 *molecular screening in medical genetics practice, we evaluated its implication in a series of patients exhibiting non-vestibular schwannomas and no *NF2 *germline alteration.

## Methods

From 1992 to 2006, 303 patients were referred from the French outpatient genetic clinics for point mutations and genomic rearrangements analysis of the *NF2 *gene to investigate the presence of schwannomas. All patients or legal representatives signed an informed consent for genetic analyses related to their disease according to the French law (Public Health Code, article R145-15-4). No specific consent was required for the present study, as the *SMARB1 *analysis is part of the genetic tests proposed to patients affected with schwannomas. Analyses were performed in a laboratory labeled by the French Biomedicine Agency ABM (lab no. 2008-gen-01). A subgroup of 56 cases exhibiting non-vestibular schwannomas as confirmed by MRI examination at time of diagnosis were selected for *SMARCB1 *point mutations screening. Medical reports were collected (number and localization of schwannomas, age at diagnosis and family history). Exons and splicing junctions of the *SMARCB1 *gene (NM_003073, NG_009303.1) were analyzed by sequencing using the SeqScape software (Applied Biosystems, Courtaboeuf France) after PCR amplification (Table 1). Functional consequence of intronic variants was evaluated using NetGene2, GeneSplicer and a splice-site model that accounts for adjacent and nonadjacent pair wise dependencies MaxEnt.

**Table 1:** Primers used for the analysis of the 9 SMARCB1 coding exons

Exon	Primer pair
1	5'CCCTCCTGATCCCTCGCAGC/5'CGGGCTACCTCGGAGCCGAT
2	5'CTGCGACCCTTATAATGAGC/5'GCGAGTGGTTTTGAAACAGG
3	5'ACCAGCAGAGTGACCCAGTG/5'AGAGATGCCCTGGCCAGGAA
4	5'GGATCAGGTCCTATACTGAC/5'AACTAAGGCGGAATCAGCAC
5	5'TTGCATACCTAGGGCTCCGG/5'GCCCGACTGCCTTGTACCAT
6	5'TGGTGCAATCTCTTGGCATC/5'TCAGTGCTCCATGATGACAC
7	5'TGGGCTGCAAAAGCTCTAAC/5'CGCTCACACAGAGAAGTCTT
8	5'ATCCACTGGGTGCCAGCAGT/5'TCTGCCTGGAAAGCCAGGTG
9	5'CCCTGTAGAGCCTTGGGAAG/5'GCCTCTGTCCTTGCCAGAAG

## Results

Thirty-one females and 25 males were included in the study. The age of onset ranged from 2 to 70 years. Six patients had a single schwannoma, 11 patients had 2 to 7 and 39 patients more than 10 schwannomas. Two patients had a positive family history. Nine different sequence variants scattered along the sequence of *SMARCB1 *were found (Table 2). Three were present in 12 patients, 10 in a heterozygous and two in a homozygous state indicating a complete linkage disequilibrium (Fisher exact test, p < 10^-6^). Two of them were present in dbSNP as rs2229354 (c.897G > A) and rs5030613 (c.1119-41G > A); the third change (c.1149-41G > A) was also considered as a polymorphism. The remaining 6 variants were unique: c.30del, c.34C > T and c.832C > T were expected to truncate the protein; c.500+5G > T was predicted to suppress the donor splicing site of exon 4, leading to p.Arg121_Cys167delinsSer and lacking the DNA-binding domain. Using the MaxEnt program, maximum entropy of -5.62 was computed compared to 0.87 with the normal sequence. Considering the existence of a rare long isoform, as a 51-bp extension at the 3' end of exon 4, this variant also encoded c.505G > T was expected to produce a p.Gly169X truncated protein lacking the two highly conserved regions Rpt1 and Rpt2 and the coiled-coil domain. The variant c.971_978delinsTGCTACCT implicated the highly conserved Lys_324 _and Tyr_326 _amino-acids, Tyr_326 _being involved in the coiled-coil domain and possibly in the nuclear localization signal and/or the binding of Akt. All five were considered deleterious. The transversion c.501-23T > G was not predicted to affect splicing using NetGene2 and GeneSplicer and was classified as not responsible for the disease. All five patients carrying a *SMARCB1 *mutation were sporadic patients diagnosed before 35 years of age with multiple non-vestibular schwannomas.

**Table 2:** Point variations and phenotypic description of the patients exhibiting a *SMARCB1 *variation

Patient	Exon	Nucleotide change	Expected effect	Sex	Age	Disease
N00390	1	c.30del	p.Phe10LeufsX6	F	35	11 non-vestibular schwannomas
N00837	1	c.34C > T	p.Gln12X	F	24	Multiple spinal schwannomas
N00328	4 (ivs)	c.[500+5G > T,505G > T]	p.[Arg121_Cys167delinsSer,Gly169X]	M	27	1 facial nerve
						7 spinal schwannomas
						2 meningiomas
N00106	5 (ivs)	c.501-23T > G	-	M	56	2 spinal schwannomas
N00225	7	c.832C > T	p.Gln278X	M	22	Multiple schwannomas and meningiomas
N00334	7	c.971_978delinsTGCTACCT	p.Lys324_Tyr326delinsIleLeuPro	M	30	Unilateral V-, VII-, spinal schwannomas
12 cases		c.[897G > A;986+57_986+58dup;1119-41G > A]				

## Discussion and conclusions

Five mutations were identified through the analysis of the *SMARCB1 *gene for point mutations in a series of 56 *NF2*-negative patients affected with non-vestibular schwannomas. Patients carrying a mutation presented with multiple schwannomas at a young age, *i.e. *10.2% in schwannomatosis patients [6] and 20% of patients diagnosed before 35 years of age, none with any family history of schwannomas. This proportion remains lower than that described in familial schwannomatosis [3,7,8]; however neither somatic mosaicisms nor genomic rearrangements were investigated [2,7]. Tumor samples were not available thus precluding dosage analyses and the demonstration of *SMARCB1 *and/or *NF2 *somatic inactivation [3].

Schwannomatosis is a rare condition characterized by the development of multiple schwannomas and the lack of vestibular nerve involvement. As germline *SMARCB1 *mutations have never been reported in cases of vestibular schwannomas, the presence of multiple non-vestibular schwannomas appears a good criterion to first investigate the *SMARCB1 *gene, as reported through several studies (Table 3). From our study, as mutations were found in patients diagnosed before 35 years of age, this aspect might also be considered to enlarge the *SMARCB1 *analysis first to the search for genomic rearrangements and somatic mosaicisms and second to the *NF2 *gene screening. On the other hand, the presence of vestibular lesions remains the first indication for an *NF2 *gene molecular analysis.

**Table 3:** Review of the clinical description of *SMARCB1 *mutation patients affected with non-rhabdoid tumors

Reference	Clinical context	Observation
[2]	1 familial schwannomatosis	case report, initial description
[3]	28 sporadic + 15 familial non-vestibular schwannomatosis patients	2/28 + 5/15 mutations
[4]	1 familial schwannomatosis with multiple meningiomas	case report
[5]	47 NF2-negative patients with multiple meningiomas	0/47
[9]	21 sporadic schwannomatosis patients	1/21 mutation
[10]	1 familial meningiomatosis	case report

## Competing interests

The authors declare that they have no competing interests.

## Authors' contributions

GR has set up the technique for *SMARCB1 *gene screening. TN and VB have supervised the work, helping in sequencing data interpretation. HS is the head of the laboratory. SO organized patients' recruitment and managed the integrated laboratory approach. All authors read and approved the final manuscript.

## Pre-publication history

The pre-publication history for this paper can be accessed here:

http://www.biomedcentral.com/1471-2377/11/9/prepub
